# Effectiveness of integrated care interventions for patients with long-term conditions: a review of systematic reviews

**DOI:** 10.1136/ihj-2021-000083

**Published:** 2022-06-16

**Authors:** Mohammad Hussein Housam Mansour, Subhash Pokhrel, Nana Anokye

**Affiliations:** Department of Health Sciences, Brunel University London, London, UK

**Keywords:** healthcare quality improvement, patient discharge, patient-centred care, length of stay, chronic disease management

## Abstract

To examine the effectiveness of integrated care intervention (ICI) models (stand-alone or combination of self-management, discharge management, case management and multidisciplinary teams models) targeting patients with one or more chronic conditions, and to identify outcome measures/indicators of effectiveness, we conducted a systematic review of published systematic reviews and meta-analyses. Included reviews comprise ICIs targeting adult patients with one or more long-term conditions. We searched MEDLINE, CINAHL and the Cochrane Database of Systematic Reviews: 60 reviews were included in the final analysis; 28 reviews evaluated ICIs focused on self-management, 4 on case management, 10 on discharge management and 5 on multidisciplinary teams; 13 reviews assessed multiple interventions that were labelled as complex. Across all reviews, only 19 reviews included intervention with multiple ICIs. Overall, interventions with multiple components, compared with interventions with single components, were more likely to improve hospital use outcomes effectively. Clinical/lifestyle/condition-specific outcomes were more likely to be improved by self-management interventions. Outcome measures identified could be classified into three main categories: organisational, patient-centred and clinical/lifestyle/condition-specific. The findings of this review may provide inputs to future design and evaluation of ICIs.

**Key messages**

**What is already known about this subject?**

- In many countries, integrated care interventions (ICIs) have formed the cornerstone of policy responses in response to budgetary and service pressures in healthcare.
- Through better coordination between and across diverse settings, such as primary, secondary and community settings, beside increasing personalisation and self-care, ICIs attempt to provide proper care and improve patients’ experiences.
- ICIs could reduce the pressure on health services by improving health-related outcomes and quality of care, thus reducing the need for various health-related services, including hospital admissions.
- Despite several attempts to analyse ICIs, findings from several studies suggested that combining multiple ICIs, such as self-management (SM), multidisciplinary teams (MDTs), discharge management (DM) and case management (CM), could yield better effects; nevertheless, evidence on whether these interventions can achieve the expected benefits remains scant.

**What does this study add?**

- ICIs including SM, CM, DM and MDT might be more effective in reducing hospital use as an adjunct to broader interventions.
- Including SM components in broad ICIs may improve results for other outcomes, particularly patient-centred outcomes.
- In the case of such interventions, organisational outcomes such as hospital admissions/readmissions, patient-centred outcomes such as quality of life and clinical outcomes such as haemoglobin A1c are examples of indicators that can be monitored to assess for effectiveness.

**How might this impact on clinical practice or future developments?**

- The study’s principal findings may have implications for policy initiatives in countries like England involving the design and implementation of ICIs, such as requiring multicomponent interventions to be considered when planning and executing ICIs.
- Service providers can achieve some ‘quick gains’ in reducing hospital utilisation by targeting specific patient categories/groups with multicomponent ICIs.
- Interventions like SM, on the other hand, could be used to increase personalisation and self-care.

## Background

According to WHO, long-term conditions (LTCs), also known as chronic diseases, are the leading cause of death and disability worldwide.^1^ It has been estimated that chronic diseases account for around 60% of all deaths worldwide.^1^ As a result, WHO proposed a plan for an integrated approach in 2002 to target the major risk factors for the common chronic diseases, including cardiovascular diseases, chronic respiratory diseases, cancer and diabetes.^2^ However, along with prevention, providing high-quality care for patients with LTCs is also a vital issue.

Patients with LTCs often experience fragmented care and require access to multiple health and social care settings.^3^ The gaps in coordination between caregivers and lack of personalisation and interactions with patients contributed to this fragmentation in care.^4^ Hence, providing such patients with the required care is a significant challenge. In this connection, integrated care interventions (ICIs) have been proposed to promote coordination between and within healthcare settings to improve patient’s experience and the outcomes of care in many countries, including England.^5 6^

‘Integration’ might be defined and used in various contexts, such as characterising interventions that improved care or quality assurance but did not require personnel to operate in novel ways.^7^ In this regard, integrated care might exist in multiple forms, and various interventions have been proposed. Some interventions, such as self-management (SM) aim to increase personalisation and self-care, supporting medication adherence or condition-specific education.^8^ On the other hand, other interventions such as case management (CM), multidisciplinary teams (MDTs) and discharge management (DM) form a collaborative process that includes communication between care givers themselves and with patients across different settings.^9^ Besides, these interventions aim to facilitate care along a continuum through effective resource coordination. Consequently, with their aims achieved, these interventions might reduce care fragmentation and improve outcomes on different levels.

Driven by the main aims of ICIs and programmes, there have been attempts to evaluate ICIs pilots suggesting potential effectiveness.^10^ However, the evidence supporting a fuller understanding of the scale and mechanisms of ICIs’ effectiveness is scanty,^11^ despite the existence of several randomised controlled trials (RCTs) evaluating the effectiveness of these interventions.^12 13^ Previous evidence synthesis attempts include systematic reviews and meta-analyses summarising the results of different studies.^12 14 15^ Some results across some reviews suggested that the combination of multiple ICIs, including SM, MDT, DM and CM, could produce better results. For instance, when SM was incorporated into MDT care or when individualised patient education was included in discharge planning, it showed the most promise.^14^ Despite this, the evidence remains scarce as to how effectively the new ICIs can deliver their expected benefits,^4^ and whether incorporating these interventions might produce better effects.

As a result, we undertook a systematic review of published reviews and meta-analyses to comprehensively examine the outcomes and effectiveness of four primary ICIs: SM, CM, MDTs, DM or any construct combining any of these interventions (complex interventions (CIs)).

## Methods

A systematic review of published reviews was conducted to provide evidence for decision-makers who need a synthesis of the most current and reliable data relevant to their context.^16^

### Population

We focused on male and female patients aged 18 years or over, with one or more LTC under management. We selected the most common diseases included in multimorbidity indices.^17^ LTCs included: heart conditions (eg, stroke), diabetes (type 1 and 2), renal diseases (eg, chronic kidney disease (CKD)), respiratory conditions (eg, asthma and chronic obstructive pulmonary disease (COPD)) and cancer.

### Intervention

The following interventions (for comprehensive descriptions, see online supplemental data) assessed by the included reviews or meta-analyses were examined:

10.1136/ihj-2021-000083.supp1 Supplementary data

#### Case management

Providing care through a collaborative process between one or more care coordinators or case managers and the patient.

#### Discharge management

Mainly facilitates effective transitions from hospital care to other settings.

#### Multidisciplinary teams

Teams composed of multiple health and/or social care professionals working together to provide care.

#### Self-management

Designed to provide patient support, typically via tailored education.

#### Other complex or broad interventions

Interventions with multiple components, any combination of the above.

### Comparator and outcomes

There was no restriction on the control groups included in the reviews. The reviews included different types of comparators, including usual care. As one of our objectives was to identify and describe the outcome measures relevant for ICMs, there was no restriction on the type of outcomes assessed. However, we focused on hospital usage outcomes such as admissions, readmissions, ED visits, length of stay (LoS) in the hospital and the most prevalent patient-centred and clinical outcomes assessed in the reviews for our major evidence synthesis.

## Inclusion and exclusion criteria

To be included in this study, a review needed to be published in the English language and meet the following requirement: (a) a systematic review that examined the effectiveness of four ICMs focussing on patients with one or more LTC in the last 10 years or (b) a meta-analysis combining effect sizes from various studies quantitatively. The following criteria was used to identify a systematic review: (i) includes a clearly established set of objectives for the investigations with predefined eligibility criteria; (ii) includes a clear description of methods with a systematic search through different databases; (iii) includes a review of findings with an assessment of the validity of the included studies and (iv) includes a systematic presentation, and synthesis, of the characteristics and findings of the included studies.

A systematic review or meta-analysis that did not meet the PICO was excluded. Reviews that included populations such as adolescence or children or populations with conditions other than the conditions of interests (eg, mental health) were excluded. Reviews that focused on interventions other than the interventions of interest were also excluded (eg, chronic care models, collaborative care models). We only included interventions that showed potential when combined, such as MDT, SM and DM. We excluded collaborative care models, given their focus on targeting patients with depression, anxiety or mental conditions.^18^ Some reviews included a variety of studies with different interventions, and thus, we only included the results that focused on the four interventions of interest in our evidence synthesis.

**Figure 1:**
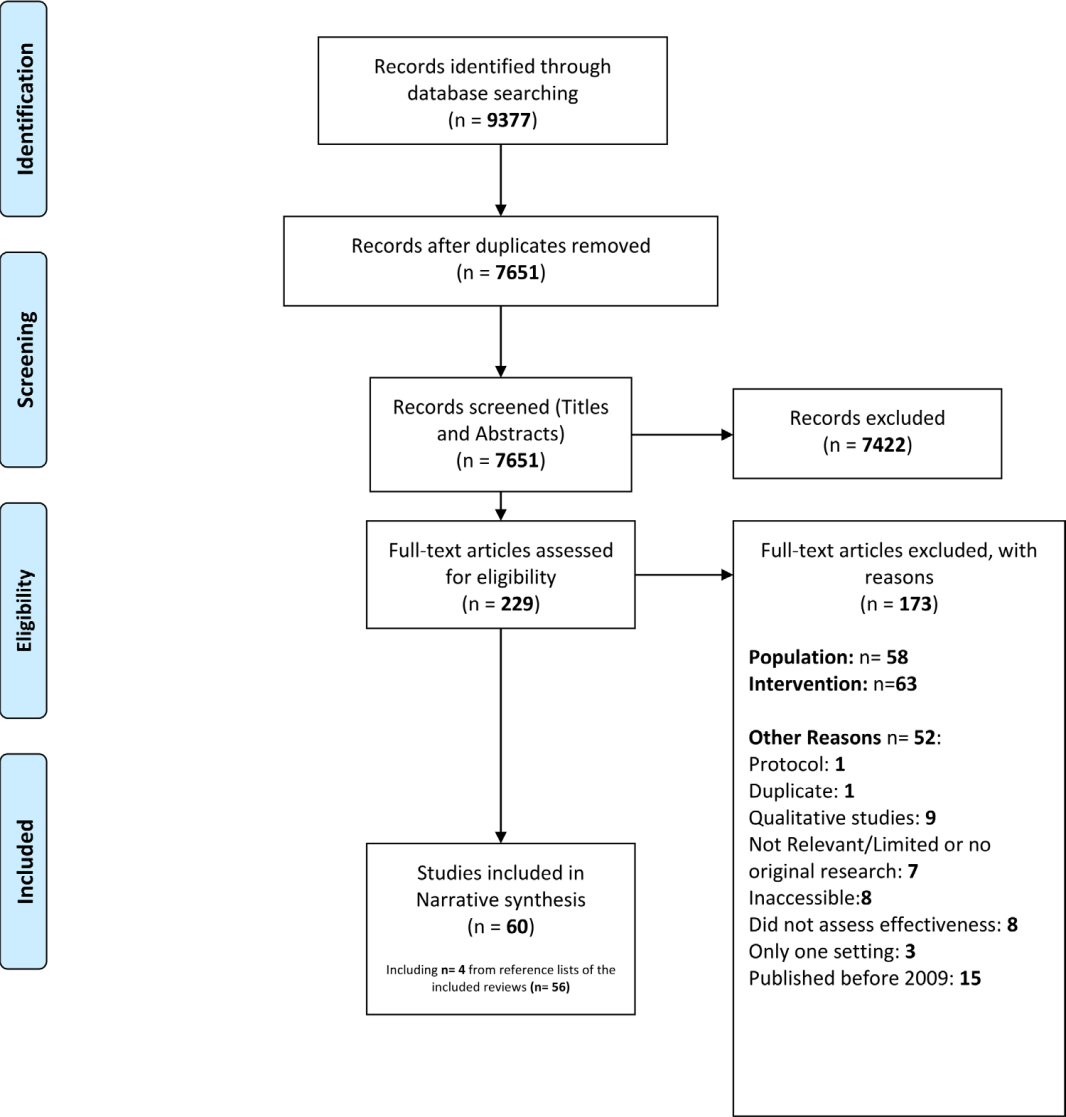
Preferred Reporting Items for Systematic Reviews and Meta-Analyses diagram of search results.

To be considered as crossing between settings, the intervention needed to be delivered simultaneously by medical personnel or caregivers within a community (eg, social care settings), acute (eg, general practitioner (GP) surgery) and/or secondary care settings (eg, hospital). Hence, studies that included interventions that did not cross the boundary between at least two health and/or social care settings were excluded. Finally, we excluded studies for other reasons such as accessibility or year of publication (figure 1).

### Search strategy

A search strategy involving a combination of keywords, informed by PICO and scoping review, was used (see online supplemental data). This strategy was deliberately broad to cover the breadth of the literature. Three electronic databases (MEDLINE, CINAHL and the Cochrane Database of Systematic Reviews) were searched for systematic reviews and meta-analyses between 2009 and 2019. The search was conducted from August to September 2019.

### Eligibility assessment, data extraction and data analysis

Search results were collected into a single reference manager software (Refworks). Titles and abstracts were screened for inclusion. In case of doubt regarding the inclusion of the study, the full text was screened for eligibility. Eligible studies had their data extracted by a single reviewer (MHHM).

Due to the vast number of reviews retrieved and the high number of outcomes and heterogeneity, the evidence synthesis was limited to a narrative review of interventions and outcomes. For the same reason, we summarised the effects of most common hospital use outcomes by intervention using the criteria presented in table 1, which consists of three main categories: positive association, negative association and no association or mixed findings. Graphs were used to summarise effects on other outcome categories. Our evaluation was fully outcome-based.

**Table 1:** Criteria adopted to report effect by outcome

– (Negative association)	Review reported statistically significant decrease in outcome measures (meta-analysis). At least half of the studies included in a review reported a significant decrease outcome measure (narrative).
+ (Positive association)	Statistically significant increase in outcome measures (meta-analysis). At least half of the studies included in a review reported a significant increase in outcome measure (narrative).
= (No association)	No significant difference reported (meta-analysis). At least half of the studies included in a review reported no significant difference (narrative). Mixed findings reported in a review with equal or slight difference between number of studies reporting negative, positive or no association (narrative). Low-quality evidence due to issues with bias, follow-up and heterogeneity.

### Quality assessment

The quality of studies was appraised using the Centre for Evidence-Based Medicine tool for critical appraisal of systematic reviews. Generally, the tool is composed of five questions: (i) clarity of the research question(s), (ii) clarity and appropriateness of search methods and likelihood of missing some studies, (iii) clarity and appropriateness of the inclusion criteria; (iv) discussion of heterogeneity and its reasons and (v) issues related to quality assessment including the use of the appropriate quality assessment tool (see online supplemental materials). The tool gives a score of one on each question and scores each review overall from 0 to 5, where 5 is the highest score which implies the highest quality.

While the first author (MHHM) applied the checklist to all included reviews, another author (NA) independently applied the same tool to 10% (n=6/60) of the included studies. The chosen studies for the second author to review included a wide breadth of scores given by the first author for representativeness reasons (3, 3.5 (two studies), 4, 4.5 and 5). Both reviewers independently agreed on the scores given to this sample of included studies.

## Results

A total of 9377 potentially eligible articles were identified (figure 1). Two hundred thirty-three articles were eligible for the full-text assessment following titles and abstracts screening. One hundred seventy-three articles were excluded following a full-text assessment for not aligning with the PICO and for other reasons (figure 1). Sixty studies matched the inclusion criteria and were included in our review. We have summarised the characteristics of these reviews in online supplemental table 1.

Around half were published between 2017 and 2019 (n=32) among the included studies, while other studies ranged between 2009 and 2016. Forty-nine reviews specified patient numbers (total 1 057 251; median 5735; range 835–277100), but across the 11 that did not, 2 studies only specified the range or the median. Seven reviews did not specify follow-up duration for their included studies, but across the 53 that did, follow-ups ranged from 2 days to 15.9 years, with most lasting up to 6 or 12 months. Additionally, 41 reviews included meta-analysis, and 19 were narrative syntheses. Three of the 41 meta-analysed reviews were systematic reviews of reviews, and four of the narrative reviews were systematic reviews of reviews.Three of the 41 meta-analysed studies and four of the 19 narrative studies were systematic reviews of reviews.

Patients with LTCs, multimorbidity or complex needs were the most commonly studied (n=29), followed by patients with COPD (n=12), patients with diabetes (n=8) and patients with heart conditions including stroke, heart failure (HF) and myocardial infarction (n=6). Patients with CKD (n=3), cancer (n=1) and asthma (n=1) were the least commonly studied. In most reviews (n=44), usual care was the comparator. Other reviews included the absence of an intervention, other interventions or attention controls as comparators. Five reviews did not specify their comparator.^12 13 19–21^

Reviews included studies with various study designs, including observational, quasi-experimental and predominantly RCTs. The reviews included assessed effectiveness by outcomes. We did not find reviews focusing on evaluating the effectiveness of interventions of interest combined. The included reviews focused on patients with different conditions and included studies assessing effects on specific groups. Evaluations of effects on general populations with different LTC in all settings simultaneously were not reported.

### Quality of the included reviews

Most of the included reviews scored 4/5 (n=18), followed by 4.5/5 (n=15) and 3.5/5 (n=9). Thirteen reviews had a full score, while only five studies scored 3/5. The overall quality of studies was high, with a mean quality assessment score (QA) of 4.2/5. The mean QA score across the interventions’ categories ranged from 4.2 (SM) to 3.75 (CM), 4.25 (DM), 3.5 (MDTs) and 4.46 (C). Overall, studies lost points on the likelihood of missing relevant studies as they either restricted their search to few databases or their search did not include a search of reference lists from relevant studies. In addition, in some of these studies, heterogeneity and its possible reasons were not explored.

### Characteristics of the intervention models

Twenty-eight studies focused on SM interventions, while 4 were purely CM, 10 focused on DM, 5 focused on MDTs and 13 were labelled as complex. Among the 13 reviews labelled as complex, 5 included studies that assessed the effectiveness of different interventions separately, including SM, CM, DM and MDTs. On the other hand, 8 reviews among the 13 labelled as complex assessed the effectiveness of a combination of different ICIs.^14 22–28^

Thirty-eight studies assessed interventions that crossed the boundary between three settings, including primary, secondary and community. On the other hand, 13 reviews assessed interventions that crossed the boundary between primary and community settings, while the rest were between secondary and community or primary and secondary.

Interventions assessed by reviews were heterogeneous. The heterogeneity was confined to three main dimensions: components, mode of delivery and personnel delivering or facilitating the support. However, interventions across each category shared common characteristics. For instance, across the 28 studies that assessed SM interventions’ effectiveness, most interventions included one or more of the following components related to disease management: action plans, goal setting, decision-making, self-monitoring, self-efficacy and problem-solving. Also, the educational components of the interventions varied with the target population. However, the educational programmes included two or more of the following components: disease general education, medication (eg, inhaler usage techniques, insulin injection) and lifestyle (eg, exercise, smoking cessation). The mode of delivery of interventions included individual or group-based, delivered face-to-face and/or via telephone with follow-up. On the other hand, personnel mainly were healthcare professionals (HCPs), which mainly included pharmacists, nurses and physicians. Pharmacists and nurses were either working as members of an MDT or in a pure pharmacist-led or nurse-led interventions.

Interventions assessed by the four reviews in the CM category were characterised by the inclusion of case managers responsible for delivering and coordinating services following care plans. Two reviews included studies with interventions characterised by the inclusion of SM components such as education and DM components such as transitional care services.^29 30^ In addition, all four reviews included CM interventions which were delivered by nurses, social workers, nurse practitioners, pharmacists and GPs who were either a member of an MDT or acted independently. Home visits and telephone follow-ups were standard service delivery components in the four reviews.

Among the 10 reviews that assessed DM interventions’ effectiveness, 5 reviews included studies with postdischarge interventions.^31–35^ Those interventions consisted mainly of ‘hospital at home’ support which included plans to manage patients’ conditions following discharge from the hospital. The plans primarily consisted of the following components: home visiting, symptom management and rehabilitation services delivered by HCPs who were either members of an MDT or acted independently. Four reviews included different transitional care interventions, which consisted of predischarge and postdischarge support with discharge planning, predischarge patient-centred instructions and postdischarge care with different forms of contact including in patient’s home or clinic visits and telephone.^36–39^ Among these five reviews, three reviews included interventions with additional SM components, including education and patient empowerment.^36 37 39^ Moreover, one review included interventions that consisted of both SM and CM components in addition to DM as a primary intervention.^40^

Among the five reviews that assessed MDTs interventions’ effectiveness, one review included MDTs interventions with additional DM components, including discharge planning.^41^ Moreover, the interventions in this review included hospital-initiated tailored exercise programmes followed by home visits and telephone follow-ups. Besides, one review included MDTs intervention, which consisted primarily of medication review and optimisation and educational counselling and SM components.^42^ One review included interventions to provide formalised links between primary and specialist care with education, medication review and SM components, including physical activity, lifestyle counselling and self-care.^19^ Finally, the same author conducted a review that included MDTs interventions focusing on information management and relational continuity, and SM support.^43^

Eight reviews across the 13 reviews which were labelled as complex included interventions with multiple subinterventions with two or more combination of MDTs, CM and SM. Only one review out of these reviews included discharge planning in addition to CM and MDTs as primary interventions.^28^ The other remaining reviews included studies with primarily individual interventions.

### Outcomes and indicators of effectiveness

The outcome measures included in the reviews are summarised in table 2. Those were divided into three main categories. Across the included reviews, there was a variation in labelling the outcome measures as primary or secondary. Some reviews considered organisational outcomes such as hospital admissions as primary outcomes. In contrast, other reviews, particularly those that assessed SM interventions, focused on clinical or patient-centred outcomes such as quality of life (Qol) or haemoglobin A1c (HbA1c) as primary outcomes. Moreover, some reviews included a mix of different outcomes with or without specifying order or focus. Note that outcomes such as depression and anxiety were labelled as patient-centred. Those were assessed in reviews as general and not clinical outcomes (given that we excluded reviews focusing on mental conditions/illnesses).

**Table 2:** Outcome measures included in the reviews

Organisational	Clinical, lifestyle and condition-specific	Patient-centred
Condition-related hospital admissions All‐cause hospital admissions Risk of admission Hospital readmissions 30 days readmissions Risk of readmission Unplanned admissions Time between discharge and readmission All‐cause mortality Condition-related mortality ED visits LoS (home) LoS (hospital) Primary care consultations Nursing home admission GP visits Social worker visits Nursing visits Outpatient visits Ambulance calls Living in an institutional setting Clinician contact	Condition-related knowledge HbA1c BMI Foot care Self-monitoring of blood glucose Dyspnoea COPD exacerbations Pulmonary functions Distance on 6 min walk Exercise capacity Courses of oral steroids Progression to ESRD Change in proteinuria excretion Risk of dialysis eGFR BP Creatinine CRP BGL Cholesterol HDL LDL Triglycerides Smoking status Physical activity Behaviour change Alcohol Diet and nutrition Cognitive function Asthma exacerbations Asthma severity score Asthma-specific Qol	Qol Self-assessed health status HRQol Subjective health status Patient satisfaction Self-efficacy Self-care behaviour/activities Risk perception Trust of physician Physical dependency Activities of daily living or extended activities of daily living Decision quality Medication adherence Depression Anxiety Fatigue

BGL, blood glucose levels; BMI, body mass index; BP, blood pressure; COPD, chronic obstructive pulmonary disease; CRP, C reactive protein; ED, emergency department; eGFR, estimated glomerular filtration rate; ESRD, end-stage renal disease; GP, general practitioner; HbA1c, haemoglobin A1c; HDL, high-density lipoprotein; HRQol, health-related quality of life; LDL, low-density lipoprotein; LoS, length of stay; Qol, quality of life.

## Effects by intervention type and outcomes

**Figure 2:**
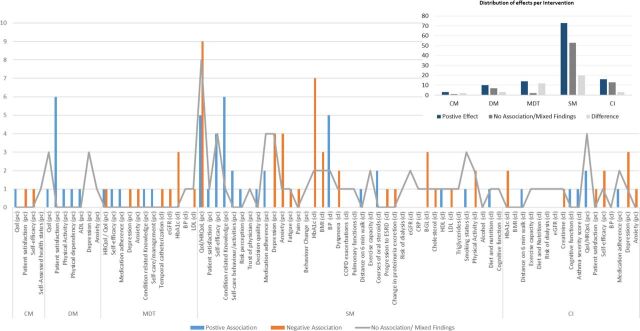
Effects by intervention on patient-centred and clinical, lifestyle and condition-specific outcomes.

Table 3 and figure 2 summarise the effects of interventions on hospital use, patient-centred and clinical outcomes. We only included the most common hospital use outcomes in table 3. Studies in bold indicated reviews which included interventions with multiple components (eg, MDT+DM+SM).

**Table 3:** Effects by intervention on hospital use outcomes

Study	Admissions	Readmissions	ED visits	LoS (hospital)
*Case management*				
Oeseburg *et al* 51	=		=	=
**Joo and Huber** 30		–	=	=
**Joo and Liu** 29		–	–	=
*Discharge management*				
Echevarria *et al* 33		–		–
**Langhorne and Baylan** 31		=		–
Gonçalves‐Bradley *et al* 35		=		
**Yang** ***et al*** 34		–		
Shepperd *et al* 32	=			=
**Braet** ***et al*** 36		–	–	
**Prvu** ***et al*** 39		=	–	–
Roper *et al* 38		–		
**Allen** ***et al*** 37		=		=
**Leppin** ***et al*** 40		–		
*Multidisciplinary teams*				
**Hickman** ***et al*** 41		–	–	–
Shi *et al* 52	–			
**Health Quality Ontario** ^43^	=		=	
**Health Quality Ontario** 19	–		–	
Self-management				
Lenferink *et al* 58	–			
Jolly *et al* 47	–			
Harrison *et al* 57	=			
Zwerink *et al* 46	–			
Zimbudzi *et al* 54	–		=	
Ditewig *et al* 49	=	=		
Wang *et al* 45	–		–	
Hosseinzadeh *et al* 50	=			
Long *et al* 44	–			
Majothi *et al* 48	=	=	=	=
Newham *et al* 55			–	
*Complex*				
**Valentijn** ***et al*** 25	–			
**Kruis** ***et al*** 27	–			–
**Kastner** ***et al*** 24				
**Takeda** ***et al*** 28	–	–		
**Peytremann‐Bridevaux** ***et al*** 26	–			
**Baker** ***et al*** 22		=		
**Mitchell** ***et al*** 23	=	–		–
Martinez-González *et al* 12	–	–	–	–
Murphy *et al* 73				
Smith *et al* 74	=			=
Baxter *et al* 13	–	=	–	–
**Damery** ***et al*** 14	–	–	=	–

ED, emergency department; LoS, length of stay.

## Hospital use

### Self-management

Most reviews that assessed SM interventions’ effect on condition-related hospital admissions reported positive results (table 3). Out of these studies, three were meta-analyses: OR 0.46, 95% CI (0.31 to 0.69),^44^ RR 0.67 95% CI (0.56 to 0.79),^45^ OR 0.57 95% CI (0.43 to 0.75).^46^ It is important to note that one review reported positive, but little effects on hospital admissions,^47^ and one reported no effect on all-cause hospital admissions.^44^ On the other hand, three reviews found insufficient evidence of SM’s effectiveness on hospital admissions,^48–50^ with two of them reporting no evidence of effectiveness on readmissions.^48 49^ Other hospital use outcomes were not frequently assessed; however, two reviews reported fewer COPD-related ED visits in the intervention group,^45^ pooled standardised mean difference (SMD)=−0.13; 95% CI=−0.23 to –0.03.^28^

### Case management

Across reviews that investigated the effectiveness of CM interventions, two reviews included studies with interventions characterised by SM components such as education, DM component such as transitional care services and MDTs intervention and the CM as a primary intervention.^29 30^ Those reviews showed the most robust evidence of a reduction in hospital readmissions in different LTC groups, particularly with interventions with multiple components. Positive effects on hospital admissions reduction were not frequently reported across studies included in all reviews.

The evidence of effects on other hospital use outcomes was mixed, with one high-quality review reporting a reduction in ED visits in five studies.^29^ Also, one review reported fewer bed days and ED visits in studies that included CM interventions with DM components.^30^ A review with mixed findings noted two factors as being essential determinants in interventions that led to positive effects on outcomes, including: (i) good communication and close cooperation between the case manager and physicians and other health professionals and (ii) the acceptance of the case manager as the coordinator for care delivery.^51^

### Discharge management

Most reviews assessing DM reported a reduction in hospital readmissions (table 3). Although some reviews with single component DM interventions showed evidence of effectiveness,^33 38^ reviews with the most evidence of effectiveness in reducing readmissions included interventions consisting of anywhere from SM, MDTs and CM with DM as the primary intervention.^34 36 40^ RR of hospital readmission in patients with different conditions reported by one of the reviews with interventions with such characteristics was 0.77 (95% CI 0.70 to 0.84). Interventions starting during hospital stay and continuing after discharge were more effective compared with interventions starting after discharge (subgroup difference p=0.02).^36^ Similarly, early readmissions were prevented by 18%, as reported by another review (RR 0.82 (95% CI 0.73 to 0.91)).^40^ In this regard, this review also reported in its exploratory subgroup analyses that interventions with many components (interaction p=0.001), involving more individuals in care delivery (p=0.05) and supporting patient capacity for self-care through SM (p=0.04) were 1.4, 1.3 and 1.3 times more effective than other interventions. DM interventions targeting patients with COPD and consisting of health education, self-management, action plan and home visits/follow-up telephone were also associated with 30% reduction in readmissions in another review (RR 0.70 (95% CI 0.63 to 0.48)).^34^

Four reviews reported no association or mixed findings concerning effects on hospital readmission.^31 35 37 39^ Among those, three included DM along with multicomponent interventions,^31 37 39^ by which one of them indicated that GP and practice nurse interventions were not effective in reducing re-hospitalisation rates.^37^ On the other hand, one of these reviews with mixed findings reported that hospital-initiated interventions seemed more effective in producing better outcomes.^39^ All reviews with multicomponent interventions which reported no association or mixed findings included only one intervention in addition to DM as a primary intervention.

Other outcomes were less frequently assessed by reviews assessing this category. However, two of the reviews including multicomponent interventions reported negative associations: ED visits: (RR 0.75 (95% CI 0.55 to 1.01)),^36^ LoS: mean difference (MD) −5.5 days (95% CI −3 to −8).^31^ On the other hand, five reviews assessed the effects on LoS at the hospital with three studies reporting a decrease in LoS,^31 33 39^ and two reporting mixed findings.^32 37^

### Multidisciplinary teams

Like the other intervention groups, MDTs showed more substantial evidence of effectiveness in combination with other components. Combining MDTs as a primary intervention with DM components showed decreased hospitalisation in patients with different LTCs.^19 41^ Other reviews with interventions holding similar characteristics showed effectiveness only in patients with COPD (admission: RR 0.67 (95% CI 0.52 to 0.87), ED: RR 0.59 (95% CI 0.43 to 0.81)), but not in patients with a history of HF.^43^ Other studies with single components showed that MDTs interventions were associated with a lower hospitalisation rate for patients with CKD, with an OR of 0.62 (95% CI 0.46 to 0.84, p<0.001).^52^ In this review, subgroup analysis showed that patients with CKD achieved better health outcomes when they received care from multiple HCPs.

### Complex interventions

Reviews that included studies assessing multicomponent complex ICIs predominantly reported substantial effects on hospital use outcomes (table 3, studies in bold). For instance, results of few meta-analyses reported a negative association in different hospital use outcomes, including hospital admissions/readmissions: admissions: OR 0.68 (95% CI 0.47 to 0.99),^24^ RR 0.38 (95% CI 0.15 to 0.95),^25^ HF-related readmissions (RR 0.64, 95% CI 0.53 to 0.78),^28^ LoS at hospitals: MD −3.78 (95% CI −5.9 to −1.67).^24^ Across the other reviews, there was evidence of better effects of interventions when combined. For example, one review reported DM with postdischarge support as the most effective intervention in reducing hospital admissions.^14^ Also, the same review reported MDTs interventions as more effective with teams that include condition-specific expertise, specialist nurses and/or pharmacists. Besides, the same review reported that SM interventions were more effective as an adjunct to broader interventions.

## Patient-centred and clinical, lifestyle and condition-specific outcomes

Reviews that assessed SM interventions were the most common in evaluating effects on these categories (figure 2). SM interventions also showed the highest number of positive effects counts in terms of effects. Although studies evaluating these interventions had the highest number of counts, the discrepancy between positive benefits and no association/mixed findings counts were the highest among this intervention category. Qol/HRQol, patient satisfaction, self-efficacy, condition-related knowledge, HbA1c, BMI, BP, depression and anxiety were the most common outcomes assessed. Results across these outcomes showed a positive increase in condition-related knowledge in all reviews except one review. Statistically significant effect sizes (SMD and MD) among studies which conducted a meta-analysis were: SMD: 0.58,^46^ 0.69^53^ and MD: 2.18.^45^ Most reviews reported a positive effect on HRQol (9 out of 11). With this in mind, statistically significant effect sizes (SMD and MD) for this outcome among studies that conducted a meta-analysis ranged between −2.69 and 0.11 (SMD) in six studies,^44 54–58^ and between −3.51 and 3.84 (MD) in three studies.^46–48^ Note that effect sizes reported for HRQol were positive effects regardless of being positive or negative in magnitude depending on the questionnaire used to measure the HRQol. Seven reviews out of nine reported a positive effect on HbA1c among patients with diabetes. Statistically significant effect sizes among reviews which pooled their results were: SMD: −0.22,^59^ and 0.11,^57^ WMD: −0.38,^60^ MD: −0.5,^54^ −0.68^61^ and −0.71.^21^ Other reviews found mixed results on the primary and other outcomes in this category, yet they were all included in the summary as shown in figure 2.^62–71^

Patient-centred and clinical outcomes were less commonly assessed with studies assessing other interventions. Among studies that assessed these outcomes, positive findings were more evident in reviews that conducted a meta-analysis. For instance, MDTs showed effectiveness in reducing HbA1c: MD −1 (95% CI −1.27 to −0.73),^43^ MD −0.55 (95% CI −0.65 to −0.45)^42^ and COPD HRQol: MD −4.05 (95% CI −6.47 to −1.63).^43^ Results across other outcomes varied; most evaluations found greater patient satisfaction, including all reviews that looked at DM and two that looked at CM and MDT as primary interventions.^19 72^ Across the complex category, reviews assessing interventions separately reported various findings concerning patient-centred and clinical outcomes. However, positive effects of individual studies with SM interventions included in these reviews were frequently reported.^12 25 73 74^ Reviews with multiple components ICIs with SM components had the most favourable effect on these outcome categories in general.^24 26 27 42 43 75^

## Discussion

The primary objective of this review was to examine the effectiveness of four ICIs in improving outcomes of care for patients with chronic conditions. Although the included reviews were heterogeneous in terms of study characteristics, our review found some evidently positive trends. SM interventions showed more positive effects in improving patient-centred outcomes, especially condition-related knowledge, HRQol and HbA1c. Other interventions showed better effectiveness in reducing hospital use when combined rather than existing as individual interventions.

Our secondary objective was to identify outcome measures to evaluate ICIs. As a result, we were able to identify various outcomes classified into three main categories: organisational, patient-centred and clinical/lifestyle/condition-specific.

### Effectiveness

The main finding of our review regarding the effectiveness of the four ICIs is that they might be more effective in reducing hospital use as an adjunct to broader interventions. In addition, including SM components might produce better results across other outcomes, especially patient-centred outcomes. Although there are some evidently positive trends regarding the effectiveness of these interventions as separate models or with specific combinations, the combination of the four might be more effective, and three main points can explain this.

First, the overall effects of all interventions varied across the primary outcomes assessed (figure 2); however, reviews that included multicomponent ICIs predominantly accounted for the most favourable outcomes when examined per review (table 3, studies in bold). The trend was visitable across all intervention groups. The CIs category included the most interventions with combined components. Hence, this trend was more noticeable across this group.

Second, the components of a broad intervention consisting of two or more of these interventions might influence effectiveness. In other words, combining specific interventions seemed to be more effective with certain hospital use outcomes. For instance, across all reviews which included multiple components ICIs,^14 19 22–31 34 36 37 39–42^ there was uncertainty in evidence regarding hospital readmissions/admission in interventions which did not combine CM, MDT or SM interventions or components.^22 31 37 39 43^

While combining multiple ICIs might produce better effects, including higher numbers of combinations could also be more effective. Not all interventions with combinations of two ICIs produced effects in reducing hospital use. On the other hand, interventions consisting of three ICIs or primary interventions with more than two components of other interventions reduced hospital use across different outcomes.^28 36 40^

Third, the main aim of ICIs in reducing fragmentation of care for patients with LTCs might be more feasible to achieve with the combination of multiple interventions or components. This can be explained by the fact that patients with LTCs often require a care plan with multiple elements. In other words, patients with chronic conditions might experience different health-related incidences which cannot be confined in terms of care in one intervention. Combining DM, MDT and CM showed evidence in reducing hospital use, while SM interventions were the highest in producing results on the patient-centred and clinical level, either as individual interventions or as part of broader interventions. Accordingly, adding an SM component could increase personalisation and self-care abilities and better affect other levels when combined with the other interventions.

An example that could explain this point is the healthcare for patients with COPD, which might range from simple medication and symptom management to hospitalisations. While an SM model might provide benefits in terms of, for instance, lifestyle management (smoking cessation, physical exercise), patients with COPD might experience a health-related incidence that might require hospitalisation. As a result, following hospitalisation, a discharge planning element combined with postdischarge SM elements rather than SM alone following discharge might increase the chance of preventing readmission. However, it can still be argued that an SM model can prevent admission in the first place, and this can be explained by the results presented in table 3 and figure 2.

Nevertheless, the aim of complete prevention of admissions is not feasible to achieve. This argument can also be applied to other interventions. For instance, patients with CKD were shown to achieve better health outcomes when they received care from multiple HCPs.^49^ As a result, an additional MDTs model to a broad intervention might increase the chance of achieving better outcomes. Given the variability across health-related incidences, which requires different forms of care, an intervention that could target multiple health-related scenarios across different settings and conditions is required. As a result, a combination of the four ICIs might be able to provide the required coverage. This model could consist of a CM component with case managers providing care plans to an MDT team which would intervene guided by other interventions (SM and DM) across different settings (hospital, home, clinic).

Achieving a model of care that can address every patient’s need and reduce care fragmentation can be difficult. Hence, in addition to combining different ICIs, it is essential to consider the components of these interventions and modes of delivery. While there was a notable variation in components and characteristics in all interventions across the assessed reviews, specific characteristics and components were reported as being more effective. DM interventions frequently existed with postdischarge interventions and hospital-initiated interventions continuing after discharge. Interventions involving more individuals in care delivery and supporting patient capacity for self-care were more effective in reducing readmissions.^37 39 40^ Moreover, there was evidence of better effectiveness of DM with postdischarge support with SM components in addition to MDTs interventions as more effective with teams that include condition-specific expertise.^14^

Some interventions cannot exist without some components by default. For example, MDTs are often characterised by focusing on different areas of action, such as case management and health education.^76^ While those are considered as central components of other interventions such as SM or CM, the focus on including components rather than complete interventions with specific characteristics combined could alter the aim of reducing care fragmentations and produce fewer results. Combinations of interventions can establish the necessary coverage of certain additional characteristics needed for specific ICIs (eg, DM), such as the involvement of more individuals in care delivery (covered by MDTs) and supporting patient capacity for complete self-care (covered by SM).

### Outcome measures

Our secondary objective in this review was to identify potential indicators to measure the effectiveness of ICIs. We were able to identify a variety of outcomes. With this in mind, these indicators could vary in relation to the type of intervention of being group/condition-specific or targeting general populations with different LTCs. In this regard, while patient-centred and clinical outcomes might serve as good indicators in the case of group/condition-specific interventions, hospital use and other organisational outcomes might be able to capture effects on the population level.

### Research implications and relevance to policymakers

The major findings of this study could have ramifications for policy initiatives aimed at reducing care fragmentation and hospital use. Integration has become a fundamental part of England’s growing healthcare policy landscape, and there are high expectations that integrating care would result in significant gains. Between 2015 and 2018, the National Health Service in England launched its vanguard programme (VP) with different ICIs focusing on general populations and care homes and aiming to provide care in different settings rather than in hospitals alone.^6^ The logic for the VP and the interventions that were shown to be beneficial in this review have a lot in common. Combining the four ICIs and providing multidisciplinary care through elements such as discharge planning and CM can enhance outcomes and reduce hospital use. Policymakers involved in implementing and designing such models can build around these findings.

Population-based interventions were not an area of focus in the included reviews. While the effects of ICIs at the population level are not yet fully understood, population-based ICIs might require specific interventions that can operate and achieve results within specific groups or conditions, especially in care homes. On the one hand, this suggests that service providers can achieve some quick gains by directing combinations of interventions like MDT, DM, CM and to specific patient categories for whom there is clear evidence of reduced hospital utilisation. On the other hand, interventions such as SM could also be implemented to maximise personalisation and self-care.

### Strength and limitations

Assessing a variety of outcomes and interventions with the inclusion of a high number of reviews is a major strength of this study. To our knowledge, this review is the first of its kind with such broad coverage. The findings of this study might provide an assumption about the effectiveness of ICIs that policymakers involved in planning and implementing might find helpful. With this in mind, another strength that might support the first point’s potential consequence is that we did not limit our search to any specific context. As a result, our review provided a better opportunity to gather evidence from a global perspective.

Even though our review had many strengths, it also had a few limitations. Our review synthesised evidence narratively, even though the quality of the included reviews ranged from moderate to high. As a result, the findings of this study should be regarded with caution. Furthermore, as previously stated, we did not attempt to investigate or draw conclusions due to the nature and purpose of our evaluation. Furthermore, we did not attempt to assess or draw conclusions regarding the methodologies used to measure effectiveness or the specific contexts in which the interventions were implemented due to the nature and purpose of our study. Lastly, we did not attempt to evaluate the efficiency or cost-effectiveness of the models under consideration.

## Conclusion

The effectiveness of four integrated care models in improving a variety of outcomes in patients with chronic diseases is highlighted in this review. Multiple-component interventions were more likely to be effective, particularly in reducing hospital use. Organisational outcomes such as hospital admissions/readmissions, patient-centred outcomes such as Qol and clinical outcomes such as HbA1c are outcomes that can be measured in the case of such interventions. While initiatives to integrate care across England’s health and social care systems have received much attention, policymakers could see better results in reducing hospital use outcomes and improving personalisation of care by considering multicomponent interventions when planning and implementing ICIs.
