# Supplementary material for: Effectiveness of integrated care interventions for patients with long-term conditions: a review of systematic reviews
Source: Integr Healthc J. 2022 Jun 16;4(1):e000083. doi: 10.1136/ihj-2021-000083 (PMC10241031; doi:10.1136/ihj-2021-000083)
Supplement: Supplementary data [file ihj-2021-000083supp001.pdf]

Included Reviews:

Supplementary Table 1: Characteristics of the included reviews.

N	Study;	Review type; Study types; n of Studies; n of Databases; n of Participants	Population(s) and Condition(s); Comparator	QA Score; Follow-up	Interventions' Summary; Settings	Main/Common Outcome measures, Primary; Secondary
Self-Management						
1	Zhao et al (2017)	SR and meta-analysis; RCTs; 20; 5; 5802	≥18 years old with T2DM; Usual Care	4.5; 24 weeks-2 years	Education programs with multiple different forms of education and SM theories including empowerment, health belief model (Behavioural), and health motivation; Primary, Community	Qol; HbA1c, BMI, self-efficacy, self-care activities, diabetes knowledge
2	Galdas et al (2015)	SR and meta-analysis; RCTs; 49; 1; N/A	Adults, 18 years or older, diagnosed with one or more LTC; Males receiving SM intervention to Females or mixed-gender groups	3.5; N/A	Distinct SM support interventions with the following elements: Physical activity, education, peer support, and HCP monitoring and feedback all delivered by different HCPs; Primary, Community	HRQoL; depression, anxiety, fatigue
3	Saheb et al (2017)	SR and meta-analysis; RCTs, 1 quasi-experimental study, and 2 observational; 20; 16; 2296	≥18 years old with T2DM; Usual care and other interventions (Except for the two observational studies)	4.5; N/A	Decision aid support presented by personalised patient information on cardiovascular risk factors ("Statin Choice"), and description of five anti-hyperglycemic drugs, their treatment burden, and impact on HbA1c in facilitating SDM regarding diabetes treatment ("Diabetes Medication Choice"); Primary, Secondary, Community	Qol; HbA1C, diabetes knowledge, decision quality, risk perception, patient satisfaction, trust of physician, medication adherence
4	Lenferink et al (2017)	SR and meta-analysis; RCTs; 22; 7; 3854	Adults diagnosed with (COPD); Usual Care	5; 2 days- 12 months	Action plans for exacerbations of COPD within a self-management intervention delivered via face to face sessions with up to 12 months with follow-ups via telephone; Primary, Secondary, Community	HRQoL, Respiratory-related hospital admissions; All-cause hospital admissions, All-cause mortality, Respiratory-related mortality, Dyspnoea, COPD exacerbations, Courses of oral steroids
5	Jolly et al (2016)	SR and meta-analysis; RCTs; 193; 8; Ranged from 10 to 743	Adults diagnosed with (COPD); Usual Care or control	3.5; 1 to 27 weeks	SM interventions with different educational components, including exercise, breathing technique and management of dyspnea, general education and other components.	HRQoL, hospital admissions

			receiving no intervention		Primary, Secondary, Community	
6	Peng et al (2019)	SR and meta-analysis; RCTs; 19; 8; 2540	≥18 years old diagnosed with (CKD)<br><br>Usual Care	5; 3-60 months	Three types of SM interventions based on Lifestyle modifications (e.g. exercise), Medical-behaviour modifications (e.g. medication adherence), and Multifactorial modifications (combinations of lifestyle and medical behaviour) all delivered face to face or/and via telehealth instruments by different HCPs including nurses;<br><br>Primary, Secondary, Community	All-cause mortality, progression to ESRD, change in proteinuria excretion, Risk of dialysis;<br><br>eGFR. BP, CRP, Distance on 6 min walk, HbA1c
7	Harrison et al (2015)	SR and meta-analysis; RCTs; 7; 7; N/A	Adults diagnosed with (COPD) receiving SM during hospitalisation for an AE-COPD or within one month of hospital discharge<br><br>Usual Care	4; 2 weeks-12 months	Comprehensive education programs delivered by nurses with face to face or/and telephone with follow-ups. Programs included topics on COPD medication management, symptoms and other related educational topics on the disease's management;<br><br>Primary, Secondary, Community	HRQoL, hospital admissions, mortality;<br><br>Knowledge, Self-efficacy, Psychologic Morbidity, Primary Care Visits, Behaviour Change, Exercise capacity
8	Howell et al (2017)	Narrative; RCTs; 55; 5; 8084	≥18 years old in the active treatment or survivorship phases of the cancer journey<br><br>Usual Care or other educational interventions	3.5; 2 weeks-12months	SM interventions (face to face with follow-up) with one or more of the core elements specified by the review which mainly included: self-efficacy and care management, coaching by a trained instructor, facilitation of uptake of health behaviours through goal setting, active development of skills to communicate with health care provider;<br><br>Primary, Secondary, Community	QoL; Fatigue, pain, depression, anxiety
9	Fryer et al (2016)	SR and meta-analysis; RCTs; 23; 1; 1863	Adults with stroke<br><br>Usual Care or other interventions	5; 4 weeks- 6 months	SM interventions with multiple components including goal setting, decision-making, self-monitoring, and problem-solving delivered face-to-face or/and via telephone with follow up;<br><br>Primary, Community	QoL; Self-efficacy
10	Zwerink et al (2014)	SR and meta-analysis; RCTs; 14; 13; 3189	Adults with COPD<br><br>Usual Care	5; 2-24 months	SM interventions with multiple components including smoking cessation and diseases knowledge, advice about exercise, and action plans delivered face-to-face or/and via the telephone with follow-up;<br><br>Primary, Secondary, Community	HRQoL<br><br>All-cause mortality, All-cause hospital admissions, Respiratory-related hospital admission, Dyspnoea, Exercise capacity, Courses of oral steroids

11	Panagioti et al (2014)	SR and meta-analysis;<br>RCTs;<br>187;<br>9;<br>N/A	≥18 years old with LTC<br><br>Usual Care or other interventions	4;<br>3-84 months	Different SM interventions with different components to improve disease-specific education, medication adherence and other disease-related management components. Delivered face-to-face or/and via telehealth instruments such as telephones;<br><br>Primary, Secondary, Community	Qol, Hospital use
12	Van Eikenhorst et al (2017)	SR and meta-analysis;<br>RCTs;<br>24;<br>6;<br>3,610	Adults diagnosed with diabetes excluding gestational diabetes<br><br>N/A	4;<br>4-24 months	Multicomponent interventions which included diabetes education, medication, lifestyle, goal setting and SM skills and other topics delivered by pharmacists via face-to-face individual or group sessions and included home visits and telephone follow-ups;<br><br>Primary, Community	Qol; Adherence to Medication, Diabetes Knowledge;<br><br>HbA1C, BGL, BMI, Lipid profile (Cholesterol, HDL, LDL), BP, self-care
13	Jonkman et al (2016)	SR and meta-analysis;<br>RCTs;<br>47;<br>5;<br>10,596	Adults diagnosed with COPD, T2DM, CHF<br><br>Usual Care	5;<br>2-8 months	Diverse interventions with different components including goal settings and actions plans, SM of symptoms, medication management, exercise and other components. Mode of delivery was heterogeneous and included individual and group sessions, exercise sessions in addition to Telehealth instruments including internet and Telephone contacts;<br><br>Primary, Secondary, Community	HRQoL
14	Steinsbekk et al (2012)	SR and meta-analysis;<br>RCTs;<br>26;<br>5;<br>2833	Adults diagnosed with T2DM.<br><br>Routine Treatment	4.5;<br>6-24 months	Group-based education sessions with or without groups discussion and home visits delivered by one or more member of an MDT (GPs, social workers, nutritionists, nurses) and with or without telephone follow-ups;<br><br>Primary, Secondary, Community	HbA1C, fasting blood glucose, diabetes knowledge, Qol and self-efficacy.<br><br>BMI, BP, Lipid profile, Patient satisfaction, and mortality
15	Newham et al (2017)	SR of reviews and meta-analysis;<br>RCTs and Reviews;<br>26;<br>3;<br>3518	Adults diagnosed with COPD<br><br>Usual care and SM intervention with single components	4;<br>6-12 months	SM interventions with either one component of symptoms management, management of mental health concerns, management of physical activities or all three together. All delivered either by a single practitioner or multidisciplinary teams, as individual/group(s) single/multiple sessions;	ED visits, HRQol

					Primary, Secondary, Community	
16	Zimbudzi et al (2018)	SR and meta-analysis; RCTs; 8; 4; 835	Adults diagnosed with T1DM, T2DM and CKD.<br><br>Usual Care	4; 3-24 months	SM interventions with the following components: education, provider feedback, provider reminders, patient education, patient reminders, and patient financial incentives with elements that included standardised training, multidisciplinary team, peer contact, keeping logs, goal setting skills, problem-solving skills, and seeking support. All delivered by single providers or MDTs as group/individual-based sessions;<br><br>Primary, Secondary, Community	BP, GFR, HbA1c;<br><br>Self-care behaviour, hospital admissions, ED visits, HRQol
17	Ditewig et al (2010)	Narrative; RCTs; 19; 4; 4162	Adults with CHF<br><br>Usual Care	4; 6-24 months	SM intervention consisting mainly of education components accompanied by self-monitoring of physical conditions, patient diary cards or devices. Interventions were delivered by primary and/or secondary care HCPs via visits or face-to-face in care settings including hospitals;<br><br>Primary, Secondary, Community	Mortality, all-cause hospitals readmissions, CHF-hospitalization rate, Qol
18	Gorina et al (2018)	Narrative; RCTs; 20; 7; 15439	Adults with LTCs (T2DM, hypertension, and hypercholesterolemia)<br><br>Usual care and groups who only received education or physical activity as a part of an SM intervention	4; 3 months-3 years	SM interventions with multiple components including diet, physical activity, adherence to medical treatment, self-care and complication prevention and other components. Delivered by nurses or MDTs, face-to-face or/and telephone and carried out as an individual or/and group sessions;<br><br>Primary, Community	Nutrition habits, Physical activity, adherence to medication, BMI, HbA1c, BP<br>(Only T2DM related outcomes were extracted)
19	Cutler (2018)	Narrative; RCTs, Longitudinal and Qualitative; 19; 3; 2708	Adults with T2DM<br><br>Other SM interventions components	3; N/A	SM interventions delivered as group sessions with multiple components including lifestyle, physical activity, medication adherence and other components;<br><br>Primary, Secondary, Community	Self-efficacy, blood glucose, HbA1c, medication adherence.
20	Wang et al (2017)	SR and meta-analysis; RCTs; 25; 11; 13297	Adults with COPD<br><br>Usual Care	5; 2-48 months	Multiple component SM interventions (Group or individual sessions) which included topics such as smoking cessation, direction to use inhalers, advice on exercise and other	Qol, COPD-related hospital admission rate, ED visits (COPD-related), Smoking status, Pulmonary functions, Depression, COPD

					components. Interventions were delivered by different HCP, including nurses and pharmacists or MDTs.<br><br>Primary, Secondary, Community	knowledge, BMI, Mortality rate.
21	Hosseinzadeh et al (2019)	Narrative;<br>RCTs;<br>20;<br>10;<br>8998	Adults with COPD<br><br>Usual Care	3;<br>6 weeks-4 years	SM interventions with the following educational components: smoking cessation counselling; breathing and coughing skills;<br>mental health education;<br>exacerbation symptoms recognition<br>and management; improving physical activity levels; the correct<br>use of inhalers; and medication compliance, delivered face-to-face or/and via the Telephone by one or more HCP;<br><br>Primary, Secondary, Community	Qol and hospitalisation rate, COPD knowledge, Self-efficacy, Physical activity, Smoking cessation, Medication adherence and use, depression, anxiety.
22	Jeong et al (2018)	SR and meta-analysis;<br>RCTs;<br>37;<br>3;<br>5860	Adults with T2DM<br><br>Usual Care	4;<br>3 -32 months	Pharmacist-led programs consisted of information on disease and medications, adherence education, survival skills regarding hypo- and hyperglycaemia incidence, and insulin injection skills delivered as face-to-face intervention, telephone counselling, or group appointments, meetings, or education sessions;<br><br>Primary, Secondary, Community	HbA1c
23	Long et al (2019)	SR and meta-analysis;<br>RCTs;<br>10;<br>4;<br>1959	Adults with COPD<br><br>Usual care or control	4;<br>2 weeks - 24months	SM interventions with at least goal setting, motivational interviewing techniques, and COPD-related health education as components. Delivered face-to-face or/and via the Telephone by one or more HCP (Nurse, pharmacist, health coach);<br><br>Primary, Secondary	HRQoL, All-cause hospital admissions; COPD related hospital admissions<br><br>Physical activity, self-care behaviour, and mood
24	Massimi et al (2017)	SR and meta-analysis;<br>RCTs;<br>29;<br>4;<br>10162	>18 years of age with a diagnosis of LTC or multi-morbidity<br><br>Usual care	5;<br>2 weeks - 24 months	Nurse-led multicomponent SM interventions with variety of modes of delivery: face-to-face or face or/and telephone and/or nurse visits;<br><br>Primary, Community	Qol, HbA1c, BP Mortality
25	Tan et al (2019)	SR and meta-analysis;<br>RCTs;<br>18;<br>7;<br>2,307	Adults with Diabetes or/and hypertension<br><br>No intervention or	4.5;<br>2-36 months	SM educational interventions consisted of individual face-to-face counselling and included educational components on the disease, complications, medication, side effects, adherence, lifestyle changes, self-	Medication adherence

			other interventions		monitoring and self-management skills. Delivered by pharmacists or nurses;<br><br>Primary, Secondary, Community	
26	Sakakibara et al (2017)	SR and meta-analysis;<br>RCTs;<br>14;<br>4;<br>2,303	Adults with a history of stroke<br><br>Usual care, waitlist control, no intervention controls	4;<br>2 weeks-24 months	SM interventions with the following components: feedback on performance, goal setting/action planning, resource utilisation, and problem-solving delivered as an individual or/and group sessions or via telephone or telehealth instruments;<br><br>Primary, Community	Risk factors of stroke: Alcohol, smoking, cholesterol, diet and nutrition, physical activity, glucose, medication adherence, BP
27	Taylor et al (2014)	Narrative review of reviews;<br>RCTs;<br>102;<br>8;<br>N/A	Adults with LTCs<br><br>N/A	4.5<br>6 weeks-1 year	A wide variety of SM interventions with different components including action plans and goal setting, modes of delivery: face to face (Our focus), remote, tele-healthcare, web-based, and personnel delivering or facilitating the support including laypeople and HCPs;<br><br>Primary, Secondary, Community	BGL, HbA1c, BP, cholesterol, Self-efficacy, HRQol, Qol
28	Majothi et al (2015)	SR and meta-analysis;<br>RCTs;<br>10;<br>8;<br>1,466	Patients with COPD, recently discharged from hospital after an acute exacerbation;<br><br>Usual Care or other interventions	4;<br>3-52 weeks	Multicomponent SM interventions delivered before or after discharge from the hospital. **Components included:** training on medication adherence, inhaler technique, smoking cessation, nutritional advice, promoted exercise, and management of dyspnea;<br><br>Primary, Secondary, Community	Primary care consultations, hospital admissions/re-admissions, LoS (Hospital), ED visits, and mortality.<br><br>HRQoL, self-efficacy, adherence to inhaler treatment and inhaler technique, smoking behaviours, physical activity, knowledge to treat exacerbations, depression score
Case Management						
29	Oeseburg et al (2009)	Narrative;<br>RCTs;<br>9;<br>3;<br>15 746	Community dwelling patients with LTCs;<br><br>Care without a case management component	3.5;<br>10–36 months	Home visits and/or telephone calls.<br>Delivered by a case manager (nurse, social worker or nurse practitioner) who was either a member of an MDT or acted independently;<br><br>Primary, Secondary, Community	Hospital LoS, ED visits, Nursing Home admission
30	Stokes et al (2015)	SR and Meta-analysis;<br>RCTs/CCTs;<br>36;<br>6;<br>23711	Adult patients with LTCs;<br><br>Care without a case management component	4;<br>6–60 months	Community-based MDTs responsible for delivering and coordinating services; MDT care plan following a caseworker assessment, case manager constantly available to deal with problems. Delivered by a care manager, nurse, pharmacist, GP collaborating with	Self-assessed health status, mortality, healthcare utilisation (GP visits, social worker visits, nursing visits, ED visits, hospital admissions, Ambulance calls), patient satisfaction

					nurse;<br><br>Primary, community	
31	**Joo et al (2019)**	Narrative review of reviews;<br>SRs;<br>7;<br>5;<br>46572	Adult patients with LTCs;<br><br>Usual Care	4;<br>1 months – 15.9 years	Nurse-led or/ MDTs-led + case managers community- and hospital-based interventions which included assessment and planning, education, transitional services, referrals to primary or other social or health services, and face-to-face or telephone contacts for regular follow-up;<br><br>Primary, Secondary, Community	Hospital Readmissions, LoS, Nursing home admissions, ED visits, GP visits
32	**Joo and Liu (2017)**	Narrative;<br>RCTs;<br>10;<br>5;<br>7125	Adult patients with LTCs;<br><br>Usual Care	3.5;<br>6 months-5 years	Nurse-led or/ MDTs-led continuous coordinated and comprehensive care intervention for participants with chronic illnesses with the following CM services common across the studies included: transitional care services between hospitals and home or other facilities, regular home visits, regular telephone calls, individual assessment and planning at the time of hospital discharge, referral services to social support or health services, education or self-management support, psychosocial supports such as empowerment and motivational encouragement and ongoing assessment until end of intervention;<br><br>Secondary, Community	Hospital Readmissions, LoS, hospital visits;<br><br>Qol, Self-efficacy
Discharge Management						
33	Echevarria et al (2016)	SR and Meta-analysis;<br>RCTs;<br>8;<br>6;<br>1414	Adults with COPD that were recently discharged from hospital<br><br>Usual Care	4.5;<br>N/A	Hospital at Home Early Supported Discharge interventions provided by nurses or/and MDTs, which included plans to manage the patients' conditions at home and after discharge. Interventions mainly included components of home visiting, symptom management, and contacting the patient via the phone;<br><br>Secondary, Community	Mortality, readmissions
34	**Langhorne and Baylan (2017)**	SR and Meta-analysis;<br>RCTs;<br>17;<br>8;<br>2422	Adults that have been admitted to hospital with a clinical diagnosis of stroke	5;<br>3-12 months	Two main types of Early supported discharge interventions:<br>1- ESD service comprised an MDT which co-ordinated discharge from hospital, post-discharge care and provided rehabilitation and patient care at home or in a community setting.	LoS at hospital, hospital, Readmission, death, physical dependency;<br><br>ADL, Qol, depression score, Patient satisfaction

			Usual Care		2- ESD team co-ordination by which discharge home and the immediate post-discharge care were planned and supervised by a coordinated multidisciplinary team and after discharge care was subsequently handed over to existing community-based agencies who provided continuing rehabilitation and support at home;<br><br>Secondary, Community	
35	Gonçalves-Bradley et al (2017)	SR and Meta-analysis;<br>RCTs;<br>32;<br>6;<br>4746	Adults with different LTCs<br><br>Usual Care	4.5;<br>3-12 months	Early discharge hospital at home interventions by which care was provided by a hospital outreach service. In addition, some interventions care was coordinated by a hospital-based stroke team or physician in conjunction with community-based services;<br><br>Primary, Secondary, Community	Mortality, Hospital readmission, Living in an institutional setting, LoS (hospital), Patient satisfaction.
36	**Yang et al (2017)**	SR and Meta-analysis;<br>RCTs;<br>31;<br>3;<br>6715	Adults with COPD<br><br>Usual Care	4.5;<br>3-12 months	Different post-discharge interventions which including home visiting (Mainly by nurses), action plans and telemonitoring, and home base rehabilitation in addition to education;<br><br>Primary, Community	Readmission rates, mortality, Qol
37	Shepperd et al (2016)	SR and Meta-analysis;<br>RCTs;<br>16;<br>5;<br>1814	Adults with different LTCs<br><br>Inpatient care	4;<br>3-12 months	Admission avoidance hospital at home interventions which provide active treatment by healthcare professionals (care was provided by single HCPs or MDTs), in the patient's home;<br><br>Primary, Community	Mortality, hospital readmissions;<br><br>Living in an institutional setting, Patient satisfaction, LoS at home and/or hospital.
38	**Braet, Weltens and Sermeus (2016)**	SR and Meta-analysis;<br>RCTs;<br>51;<br>2;<br>10-3988 (Median 175)	Adults discharged from a medical or Surgical ward<br><br>Usual Care, attention controls, other interventions.	5;<br>4 weeks-3 months	Post or/and pre-discharge interventions categorise based on a taxonomy stated by the review: education, discharge planning, medication intervention, appointment scheduled, rehabilitation, streamlining, home visit, patient empowerment, Transition coach, patient-centred documents, timely communication. Timely follow-up, Telephone call, patient hotline, Telemonitoring.<br><br>Primary, Secondary, Community	Readmissions;<br><br>ED visits, mortality, patient satisfaction.
39	**Prvu et al., (2012)**	Narrative;<br>RCTs;<br>62;	Adults with Stroke and MI	4.5;<br>N/A	Different types of Transitional care interventions which included hospital initiated (Start at the	Mortality, patient satisfaction, Qol, Physical activity, depression, anxiety,

		4;<br>N/A	Usual Care	(We could not access the supplementary material provided by this review which included information on follow-ups)	hospital), Patient and family education, community-based, diseases management provided by single HCP or social worker or MDTs with different forms of contact including in-person home or clinic visits and telephone;<br><br>Primary, Secondary, Community	LoS (hospital), hospital readmissions, Specialist visits, ED visits, outpatient visits.
40	Roper et al (2017)	Narrative;<br>Observational;<br>3;<br>3;<br>277100	Adults with LTCs<br><br>No intervention	3;<br>11-23 months	Transitional care interventions with the following components: high-intensity service for high-risk patients, post-hospital palliative care consultations, home visits by care managers, telehealth management, pre-discharge patient-centred instructions;<br><br>Primary, Secondary, Community	30 days readmissions (by percentage and by risk groups), Risk of readmission,
41	**Allen et al (2014)**	Narrative;<br>RCTs;<br>12;<br>8;<br>4522	Adult with LTCs<br><br>Standard Hospital Discharge	3.5;<br>1-6 months	Transitional care interventions with mainly discharge planning including health teaching medication and symptoms managements (Self-management), post-discharge follow-ups via phone or in-person;<br><br>Primary, Secondary, Community	Readmission, LoS (Hospital);<br><br>Depressive symptoms, Qol
42	**Leppin et al (2014)**	SR and Meta-analysis;<br>RCTs;<br>42;<br>6;<br>8401	Older patients and patients with LTCs<br><br>Usual care and other interventions	4;<br>2 weeks-1 months	DM interventions which included anywhere from the following: CM, patient education and self-management<br>, home visits delivered by nurses or caregivers and other HCPs;<br><br>Secondary, Community	30 days readmission
Multidisciplinary Teams						
43	**Hickman et al (2015)**	Narrative;<br>RCTs;<br>7;<br>4;<br>1558	Older Patients with complex needs<br><br>Usual Care	3;<br>2-6 months	Different complex MDTs interventions with different components including standard geriatric care criteria and oral and written recommendations, hospital initiated tailored exercise program plus 1 home visit post-discharge, & phone calls for maximum 24 weeks, discharge planning (With MDT component), tailored geriatric treatment, daily multidisciplinary geriatric care all with different	Hospital readmissions, LoS (Hospital), ED visits

					modes of delivery including telephone and provided by different MDTs;<br><br>Primary, Secondary, Community	
44	**Siaw et al (2019)**	SR and Meta-analysis;<br>RCTs;<br>16;<br>3;<br>2422	Adults with T1DM/T2DM<br><br>Usual Care	4;<br>3-12 months	Medication review, medication optimisation, and educational counselling delivered by single or multiple care providers as a member of an MDT (Pharmacists, nurses, dietitians, community health workers, health coaches, and peer leaders);<br><br>Primary, Secondary, Community	BP, HbA1c, LDL<br><br>Qol, emotional distress (depression and anxiety), Diabetes-related knowledge, self-efficacy, medication adherence, self-management (defined as care activities such as diet, exercise, self-monitoring of blood glucose, foot care, and smoking cessation
45	Shi et al (2018)	SR and Meta-analysis;<br>RCTs, Cohort;<br>21;<br>5;<br>10,284	Adults with CKD<br><br>Composition of the MDT	3.5;<br>1-5.7 years	N/A	All-cause mortality, temporal catheterisation, risk of hospitalisation, eGFR
46	**Health Quality Ontario (2012)**	SR of reviews and Meta-analysis;<br>RCTs;<br>24;<br>6;<br>N/A	Adult patients with HF, diabetes, or COPD<br><br>(only COPD and HF patients were targeted with the intervention of interest-MDT-)<br><br>Usual Care	3.5;<br>N/A	Interventions to provide formalised links between primary and specialist care via disease-specific education, medication review, physical activity and lifestyle counselling, self-care and follow-up. Delivered by intermediate care teams including GPs, specialists, nurses, social workers, pharmacists, dieticians.<br><br>Primary, Secondary, Community	All-cause mortality, Hospitalization, ED visits, HbA1c, BP, cholesterol, Qol
47	**Health Quality Ontario (2013)**	Narrative;<br>Observational, RCTs and SR;<br>20;<br>5;<br>N/A	Adult patients with one or more chronic diseases<br><br>N/A	3.5;<br>N/A	Informational, management and relational continuity. Assessed by: Duration (length of relationship), Density (number of visits with the same provider in a set period), Dispersion (visits with distinct providers), Sequence (order of seeing providers).<br><br>Primary, Community	All-Cause Mortality, Hospitalization, ED visits, HbA1c
Complex Interventions						
48	Baxter et al (2018)	Narrative;<br>RCTs;<br>267 (123 quantitative, 101 qualitative, 43 reviews);	All patients including patients with LTCs<br><br>N/A	4.5;<br>1-5.7 years	Wide variety of CM and MDT interventions (In addition to ICP which was not focused on in our review);<br><br>Primary, Secondary, Community	GP appointments, Clinician contact, LoS, unplanned admissions, readmissions, ED visits, Patient satisfaction

		11;<br>N/A				
49	Flanagan, Damery and Combes (2017)	Narrative; Review of Reviews;<br>41;<br>11;<br>159,134	Adults with LTCs<br><br>Usual Care	4;<br>1-60 months	Assessed a wide variety of interventions, including SM, CM, MDT, CCM, and DM.<br><br>Primary, Secondary, Community	Ool
50	**Valentijn et al (2018)**	SR and Meta-analysis;<br>RCTs;<br>14;<br>3;<br>4693	Adults with CKD<br><br>Usual Care	4;<br>3 – ≥12 months	SM, CM, MDTs interventions and SM and CM interventions combined with MDTs;<br><br>Primary, Secondary, Community	All-cause mortality, HRQol, all-cause hospitalisation, eGFRs, risk of dialysis, BP, creatinine
51	Smith et al (2016)	SR and Meta-analysis;<br>RCTs;<br>18;<br>9;<br>8727	Adults with multimorbidity<br><br>Usual Care	5;<br>6 –12 months	SM, MDTs, and CM interventions labelled as patient-oriented or organisational, delivered mainly by nurses through visits and face-to-face with or without telephone follow-ups;<br><br>Primary, Community	Hb1Ac, BP, Cholesterol, Mortality, Depression scores, Anxiety scores, Qol, Self-efficacy, Hospital admission, Exercise/diet, self- care
52	**Kruis et al (2013)**	SR and Meta-analysis;<br>RCTs;<br>26;<br>4;<br>2997	Adults with COPD<br><br>Usual care or other interventions	5;<br>3 –24 months	Integrated Disease management interventions with multiple components (Sub-interventions) including SM, CM, and MDTs;<br><br>Primary, Secondary, Community	HRQol, Respiratory-related hospital admissions, LoS (Hospital)
53	Murphy et al (2017)	SR and Meta-analysis;<br>RCTs;<br>42;<br>4;<br>11250	Adults with poor control of T2DM<br><br>Usual care or minor enhanced elements of care	4.5;<br>3 –36 months	Organisational interventions (Included CM), and patient-centred interventions (Included SM);<br><br>The review included other single element and telehealth interventions which is not the focus of our review. Hence, we only focused on the CM and SM interventions included.<br><br>Primary, Community	HbA1c, BP<br>, lipid control<br><br>BMI, Depression, Medication adherence
54	**Kastner et al (2018)**	SR and Meta-analysis;<br>RCTs;<br>25;<br>5;<br>12 579	Older patients with multimorbidity<br><br>Usual care	4.5;<br>3 –36 months	Care coordination interventions which included combinations of multiple interventions including CM, SM, ED (Separate Education intervention with no other SM components);<br><br>Primary, Secondary, Community	Depressive symptoms, HbA1c, mortality;<br><br>Cognitive functioning, Qol
55	**Takeda et al (2019)**	SR and Meta-analysis;<br>RCTs;<br>47;<br>4;<br>10,869	Adults with a history of HF<br><br>Usual Care	5;<br>2 –12 months	CM interventions consisting of intense monitoring of patients following discharge from hospital done by a nurse and typically involves home visits or telephone calls, or both. MDTs with a holistic approach to the individuals' medical, psychosocial, behavioural and	All-cause mortality, HF-related readmissions, All-cause readmissions;<br><br>HRQol

					financial circumstances and typically involve several different professions working in collaboration;<br><br>Secondary, Community	
56	**Peytremann-Bridevaux, et al (2015)**	SR and Meta-analysis;<br>RCTs;<br>20;<br>5;<br>81,746	>16 years dragonised with asthma<br><br>Usual Care	5;<br>3 –12 months	Interventions that included three main components: Organisational targeting Patients (CM, and other elements such as structured follow-ups), Organisational targeting healthcare professional and systems (Teamwork, integration of care), Patient-centred education and SM;<br><br>Primary, Secondary, Community	Asthma-specific quality of life score, hospitalisation, ED visits, Asthma exacerbations, Self-efficacy, Asthma severity score
57	**Baker et al (2018)**	Narrative;<br>RCTs;<br>15;<br>1;<br>7813	Adults with LTCs and/or multimorbidity<br><br>No intervention	4.5;<br>1-36 months	Comprehensive care management interventions with SM and elements of CM such as care plans and care coordination with additional elements such as care navigation.<br><br>N/A	ED visits, readmissions, outpatient GP visits, HbA1c, BP, BMI, Depression score, Medication adherence
58	**Damery, Flanagan and Combes (2016)**	Narrative;<br>Review of Reviews;<br>50;<br>11;<br>219 475	Adults with LTCs<br><br>Usual Care	4.5;<br>2 weeks-60 months	Assessed a wide variety of interventions, including SM, CM, MDT, CCM, and DM.<br><br>Primary, Secondary, Community	Admissions, readmissions, LoS, ED visits
59	Mitchell et al (2015)	Narrative;<br>RCTs and Quasi-experimental;<br>14;<br>6;<br>5735 patients	Adults with LTCs<br><br>Usual Care	4.5;<br>12-24 months	The review looked at elements of different integrated primary-secondary care models which included combinations of MDTs and SM education elements with communication and information exchange, shared care guidelines or pathways as additional elements to support effectiveness.<br><br>Primary, Secondary	Clinical outcomes for diabetes, heart failure, and COPD. Readmissions
60	Martínez-González et al (2014)	SR of reviews and meta-analysis;<br>RCTs, SRs;<br>27;<br>4;<br>N/A	Patient with LTC<br><br>N/A	3;<br>3-52 weeks	Included any interventions based on disease management, case management, managed care, comprehensive care, multidisciplinary care, coordinated care, team care, CCMs.<br><br>Primary, Secondary, Community	HbA1c, BP, Exercise capacity, Qol, Patient satisfaction, Medication adherence, admissions, readmissions, LoS, Ed visits, mortality, Time between discharge and readmission.

Qol: Quality of Life, HRQoL: Health-Related Quality of Life, HbA1c: Hemoglobin A1c, BMI: Body Mass Index, ADL: Activities of Daily Living, COPD: Coronary Obstructive Pulmonary Disease, CKD: Chronic Kidney Disease, MI: Myocardial Infarction, HF: Heart Failure, T1DM/T2DM: Type 1 or 2 Diabetes Mellitus, eGFR: Estimated Glomerular Filtration Rate, BP: Blood Pressure, CRP: C-reactive protein, BGL: Blood Glucose Levels, ESRD: End Stage Renal Disease, LoS: Length of Stay, HDL/LDL: High/Low Density Lipoprotein, CHF: Chronic Heart Failure, ED: Emergency Department, GP: General Practitioner, SR: Systematic Review, CCM: Chronic Care Model, CCT: Controlled Clinical Trial, RCT: Randomised Control Trial, ESD: Early Supported Discharge, N/A: Not Applicable

Description of Interventions:

Supplementary Table 2: Intervention groupings with description (descriptions were adapted from the work of Damery, Flanagan and Combes (2016))

Category	Description of intervention
**Self-management**	Interventions designed to provide patient support, typically via tailored education to inform the patient about their condition(s), recognising signs and symptoms of disease exacerbation, dietary and lifestyle advice and/or condition-specific education supporting medication adherence
**Case management**	Based on implementation of a collaborative process between one or more care coordinators or case managers and the patient, to assess, plan and facilitate service delivery for patients with chronic diseases, particularly when transitions across healthcare settings are required
**Discharge management**	Interventions designed to facilitate effective transitions from hospital care to other settings. Typically includes a pre-discharge phase of support, transitional care for the move between the hospital and community/home setting and post-discharge follow-up and monitoring, often incorporating rehabilitation or reablement support
**Multidisciplinary teams**	Interventions comprising teams composed of multiple health and/or social care professionals working together to provide care for people with complex needs. Teams typically included condition-specific expertise, nurses, occupational therapists, physiotherapists, social workers, GPs and occasionally pharmacists or case managers
**Complex interventions**	Interventions with multiple components (Any combination of the above or reviews which assessed a range of interventions rather than focusing on a single intervention or service model

SCOPING REVIEW

SEARCH TERMS (IN SCOPUS):

(TITLE-ABS-KEY ("integrated care") AND TITLE-ABS-KEY (chronic*) AND TITLE-ABS-KEY (outcomes OR "quality of life" OR health* OR effectiveness) AND TITLE-ABS-KEY ("systematic review" OR "meta-analysis ")) AND (LIMIT-TO (PUBSTAGE , "final")) AND (LIMIT-TO (LANGUAGE , "English")).

RESULTS

Supplementary Table 3: Results of our Scoping Review

Study	Study Title	Databases	Key Search Terms
**(Flanagan, Damery and Combes, 2017)**	The effectiveness of integrated care interventions in improving patient quality of life (QoL) for patients with chronic conditions. An overview of the systematic review evidence	MEDLINE, Embase, ASSIA (Applied Social Sciences Index and Abstracts), PsycINFO, Health Management Information Consortium database (HMIC), CINAHL, Cochrane library (including the Health Technology Assessment (HTA) database, Cochrane Database of Systematic Reviews, Database of Abstracts of Reviews of Effectiveness—DARE), EPPI-Centre library, TRIP database and the Health Economic Evaluations Database (HEED)	chronic OR “long term” OR Multimorb* OR morbidit*<br><br>MeSH terms for the 11 specific chronic diseases identified from scoping searches:<br>Hypertension<br>Diabetes Mellitus<br>Cardiovascular Diseases<br>Coronary Disease<br>Stroke<br>Ischemic Attack, Transient<br>Pulmonary Disease, Chronic Obstructive<br>Neoplasms<br>Cancer<br>Depression<br>Dementia<br>Arthritis<br>Model OR integrat* OR “Case management”, “patient centred”
**(Damery, Flanagan and Combes, 2016)**	Does integrated care reduce hospital activity for patients with chronic diseases? An umbrella review of systematic reviews	Same as above (Flanagan, Damery and Combes, 2017)	Same as above (Flanagan, Damery and Combes, 2017)
**(Martínez-González et al., 2014)**	Integrated care programmes for adults with chronic conditions: A meta-review	MEDLINE, CINAHL Embase, The Cochrane Database of Systematic Reviews	Chronic*, integra*, health planning*, care, healthcare or health care, “mental disorders”, HIV, depression, disorder*
**(Ouwens et al., 2005)**	Integrated care programmes for chronically ill patients: a review of systematic reviews	Medline and Cochran	‘disease management’, ‘patient care management’, ‘patient-centred care’, ‘health planning’, ‘delivery of health care integrated’

SEARCH STRATEGY

The most common search term used by the reviews of reviews in Table 2 were selected. We selected the terms based on the PICO framework below:

Population: Patients with Chronic diseases or long-term conditions

Keywords: Chronic, long term, Multimorbidity, complex needs

Search terms: Chronic OR “long term” OR Multimorb* OR complex

Intervention: Integrated business case (Combined integrated care interventions)

Keywords: integrated care Program/intervention, complex intervention/program, multidisciplinary intervention/program.

Comparator: single integrated care interventions (Based on single models), or usual care

Keywords: Case management, Multidisciplinary teams, Self-management, discharge-management.

Search Terms for comparator and intervention: integrat* OR multidisciplinary OR management OR discharge OR comprehensive OR continuity OR collaborative OR continuum OR shared OR transitional OR “community based”

AND Program OR Intervention* OR Care OR healthcare OR “health care” OR planning

Outcome: Reduction in hospital admissions, increase in quality of care and health outcomes, increase in quality of life,

Keywords: hospitalisation, hospital care, hospital admissions, quality of life, quality of care, health outcomes.

Search Terms: hospital* OR “quality of life” OR Qol OR outcomes OR effect* OR admissions OR re?admissions OR Rehabilitation OR reduc* OR prevention

Comprehensive Search Terms:

CINAHL Plus and MEDLINE via EBSCO:

1- Chronic OR “long term” OR Multimorb* OR complex (ALL FIELDS)

2- **AND** integrat* **OR** multidisciplinary **OR** management **OR** discharge **OR** comprehensive **OR** continuity **OR** collaborative **OR** continuum **OR** shared **OR** transitional **OR** “community based” **OR** Primary **OR** “Primary care homes” OR “patient activation” OR “Patient centred” OR personalised OR personalized (ALL FIELDS)

3- **AND** Program **OR** Intervention* **OR** Care **OR** healthcare **OR** “health care” **OR** planning (ALL FIELDS)

4- **AND** hospital* **OR** “quality of life” **OR** Qol **OR** outcomes **OR** effect* **OR** admissions **OR** re?admissions **OR** Rehabilitation **OR** reduc* **OR** prevention (ALL FIELDS)

5- **AND** "systematic review" **OR** "meta-analysis" AND Review (TITLE)

6- **NOT** protocol **OR** "cost effec*" (TITLE)

Cochrane Database of Systematic Reviews:

1- Chronic OR “long term” OR Multimorb* OR complex (Title Abstract Keyword)

2- **AND** integrat* **OR** multidisciplinary **OR** management **OR** discharge **OR** comprehensive **OR** continuity **OR** collaborative **OR** continuum **OR** shared **OR** transitional **OR** “community based” **OR** Primary **OR** “Primary care homes” OR “patient activation” OR “Patient centred” OR personalised OR personalized (Title Abstract Keyword)

3- **AND** Program **OR** Intervention* **OR** Care **OR** healthcare **OR** “health care” **OR** planning (Title Abstract Keyword)

4- **AND** hospital* **OR** “quality of life” **OR** Qol **OR** outcomes **OR** effect* **OR** admissions **OR** re?admissions **OR** Rehabilitation **OR** reduc* **OR** prevention (Title Abstract Keyword)

Boolean/Phrase (EBSCO):

TX (Chronic OR “long term” OR Multimorb* OR complex) AND TX (integrat* OR multidisciplinary OR management OR discharge OR comprehensive OR continuity OR collaborative OR continuum OR shared OR transitional OR “community based” OR Primary OR “Primary care homes” OR “patient activation” OR “Patient centred” OR personalised OR personalized) AND TX (Program OR Intervention* OR Care OR healthcare OR “health care” OR planning) AND TX (hospital* OR “quality of life” OR Qol OR outcomes OR effect* OR admissions OR re?admissions OR Rehabilitation OR reduc* OR prevention) AND TI ("systematic review" OR "meta-analysis" AND Review) NOT TI (protocol OR "cost effec*")

Boolean/Phrase (Cochrane):

Chronic OR “long term” OR Multimorb* OR complex in Title Abstract Keyword AND integrat* OR multidisciplinary OR management OR discharge OR comprehensive OR continuity OR collaborative OR continuum OR shared OR transitional OR “community based” OR Primary OR “Primary care homes” OR “patient activation” OR “Patient centred” OR personalised OR personalized in Title Abstract Keyword AND Program OR Intervention* OR Care OR healthcare OR “health care” OR planning in Title Abstract Keyword AND hospital* OR “quality of life” OR Qol OR outcomes OR effect* OR admissions OR re?admissions OR Rehabilitation OR reduc* OR prevention

Databases: We selected the most common databases used by the reviews included in table 2. As a result, we decided to include: MEDLINE, CINAHL, and the Cochrane Database of Systematic Reviews.

Quality Assessment Tool

Supplementary Table 4: Centre for Evidence-Based Medicine (CEBM) tool for critical appraisal of systematic reviews

WHAT QUESTION (PICO) DID THE SYSTEMATIC REVIEW ADDRESS?	
What is best?	Where do I find the information?
The main question being addressed should be clearly stated. The exposure, such as a therapy or diagnostic test, and the outcome(s) of interest will often be expressed in terms of a simple relationship.	The ***Title, Abstract*** *or final paragraph of the* ***Introduction*** should clearly state the question. If you still cannot ascertain what the focused question is after reading these sections, search for another paper!
This paper: Yes ☐ No ☐ Unclear ☐<br>Comment:	
**F - Is it unlikely that important, relevant studies were missed?**	
What is best?	Where do I find the information?
The starting point for comprehensive search for all relevant studies is the major bibliographic databases (e.g., Medline, Cochrane, EMBASE, etc) but should also include a search of reference lists from relevant studies, and contact with experts, particularly to inquire about unpublished studies. The search should not be limited to English language only. The search strategy should include both MESH terms and text words.	The ***Methods*** section should describe the search strategy, including the terms used, in some detail. The ***Results*** section will outline the number of titles and abstracts reviewed, the number of full-text studies retrieved, and the number of studies excluded together with the reasons for exclusion. This information may be presented in a figure or flow chart.
This paper: Yes ☐ No ☐ Unclear ☐<br>Comment:	
A - WERE THE CRITERIA USED TO SELECT ARTICLES FOR INCLUSION APPROPRIATE?	
What is best?	Where do I find the information?
The inclusion or exclusion of studies in a systematic review should be clearly defined a priori. The eligibility criteria used should specify the patients, interventions or exposures and outcomes of interest. In many cases the type of study design will also be a key component of the eligibility criteria.	The ***Methods*** section should describe in detail the inclusion and exclusion criteria. Normally, this will include the study design.
This paper: Yes ☐ No ☐ Unclear ☐<br>Comment:	

A - WERE THE INCLUDED STUDIES SUFFICIENTLY VALID FOR THE TYPE OF QUESTION ASKED?	
What is best?	Where do I find the information?
The article should describe how the quality of each study was assessed using predetermined quality criteria appropriate to the type of clinical question (e.g., randomization, blinding and completeness of follow-up)	The ***Methods*** section should describe the assessment of quality and the criteria used. The ***Results*** section should provide information on the quality of the individual studies.
This paper: Yes ☐ No ☐ Unclear ☐<br>Comment:	
T - WERE THE RESULTS SIMILAR FROM STUDY TO STUDY?	
What is best?	Where do I find the information?
Ideally, the results of the different studies should be similar or homogeneous. If heterogeneity exists the authors may estimate whether the differences are significant (chi-square test). Possible reasons for the heterogeneity should be explored.	The ***Results*** section should state whether the results are heterogeneous and discuss possible reasons. The forest plot should show the results of the chi-square test for heterogeneity and if discuss reasons for heterogeneity, if present.
This paper: Yes ☐ No ☐ Unclear ☐<br>Comment:	
